# tRNA-Derived Fragment tRF-17-79MP9PP Attenuates Cell Invasion and Migration *via* THBS1/TGF-β1/Smad3 Axis in Breast Cancer

**DOI:** 10.3389/fonc.2021.656078

**Published:** 2021-04-12

**Authors:** Dongping Mo, Fang He, Junyu Zheng, Huanhuan Chen, Li Tang, Feng Yan

**Affiliations:** Department of Clinical Laboratory, Nanjing Medical University Affiliated Cancer Hospital & Jiangsu Cancer Hospital & Jiangsu Institute of Cancer Research, Nanjing, China

**Keywords:** tRF-17-79MP9PP, THBS1, breast cancer, biomarker, TGF-β1/Smad3 signaling

## Abstract

tRNA derivatives have been identified as a new kind of potential biomarker for cancer. Previous studies have identified that there were 30 differentially expressed tRNAs derivatives in breast cancer tissue with the high-throughput sequencing technique. This study aimed to investigate the possible biological function and mechanism of tRNA derivatives in breast cancer cells. One such tRF, a 5’-tRF fragment of tRF-17-79MP9PP (tRF-17) was screened in this study, which is processed from the mature tRNA-Val-AAC and tRNA-Val-CAC. tRF-17 with significantly low expression in breast cancer tissues and serum. The level of tRF-17 differentiated breast cancer from healthy controls with sensitivity of 70.4% and specificity of 68.4%. Overexpression of tRF-17 suppressed cells malignant activity. THBS1 (Thrombospondin-1) as a downstream target of tRF-17, and reduction of THBS1 expression also partially recovered the effects of tRF-17 inhibition on breast cancer cell viability, invasion and migration. Besides, THBS1, TGF-β1, Smad3, p-Smad3 and epithelial-to-mesenchymal transition related genes N-cadherin, MMP3, MMP9 were markedly down-regulated in tRF-17 overexpressing cells. Moreover, tRF-17 attenuated the THBS1-mediated TGF-β1/Smad3 signaling pathway in breast cancer cells. In general, the tRF-17/THBS1/TGF-β1/smad3 axis elucidates the molecular mechanism of breast cancer cells invasion and migration and could lead to a potential therapeutic target for breast cancer.

## Introduction

In recent years, more than 90% of non-coding RNA (ncRNAs) of genome products, including transfer ribonucleic acids (tRNA), ribosomal RNA (rRNA), microRNAs (miRNAs), small nuclear RNA (snRNA), long non-coding RNA (lncRNA), and circular RNAs (circRNAs) play a widespread and important role both inside and outside of the cell, which involved in cellular biological function and cellular metabolism (1–3). Due to increased utilization of high-throughput sequencing and microarray technologies, more types of non-coding RNAs are being identified (4, 5).

A new class of ncRNAs called tRNA derivatives are one of them, which were generated by cleavage of mature or precursor tRNAs under the particular environmental stresses (6, 7). A wealth of intriguing studies discovered that tRNA derivatives mainly were divided into two types: tRNA-derived stress-induced RNAs (tiRNAs) (8) and tRNA-derived fragments (tRFs) (9). tRNAs derivatives are not merely tRNA degradation debris but have multiple biological functions, including acting as signaling molecules in stress responses and regulators of gene expression (10, 11). Some studies have demonstrated that tRNAs derivatives are similar to miRNAs expressed in tissues and cells in various organisms (12, 13). Generally, increasing evidence revealed that a series of tRNAs derivatives is associated with several types of human diseases and could serve as effective tools for cancer diagnose and treatment.

Although decades of research have proved that metastasis at distant site is the major obstacle of treatment and the main cause of cancer mortality in breast cancer patients (14), the molecular and cellular mechanisms underlying is still poorly understood. Whether these new class of ncRNAs (tRNAs derivatives) involved in the mechanisms of breast cancer? In previous research, we used a high-throughput sequencing technique to determine differential expression of tRNAs derivatives (tRF&tiRNA) in breast cancer tissue specimens. According to the tRF&tiRNA-Seq data, we identified 17 types of tRF&tiRNA that were most up-regulated (fold change >2.6, *P <*0.05) and 13 types of tRF&tiRNA that were most down-regulated in the tumor tissues (fold change >2.0, *P <*0.05) (12, 15). tRF-17-79MP9PP (fold change = −4.8984, *P* = 0.0276) is one of the screened tRF&tiRNAs, which clinical significance and biological functions in breast cancer remain poorly understood. In this work, we detected the expression level of tRF-17-79MP9PP in breast cancer tissues, cell lines and serum samples. Meanwhile, the potential clinical significance and biological functions of tRF-17-79MP9PP and other related genes in downstream pathways were also investigated.

## Materials and Methods

### Clinical Samples

The study was approved by the Clinical Research Ethics Committee of Nanjing Medical University. From January 2016 to December 2019, we collected 76 serum samples from patients with breast cancer, who visited the thoracic surgery department of Jiangsu Cancer Hospital. These patients with breast cancer had not received any treatments before collection of serum samples. Breast cancer tissue samples and matched non-tumor adjacent tissues (NATs) were also obtained from patients and stored at −80 °C until further processing. Pathological classification, grading, and staging were according to WHO Classification of Breast Tumor, 2012, differentiation status of cancer cell, and TNM system (16). Furthermore, age-matched control serum samples were obtained from 27 healthy women. Twenty patients with benign breast diseases including hyperplasia, fibroadenoma, and breast nodule were also collected. The general characteristics of study subjects were displayed in **Supplementary Table 1** (serum samples) and **Supplementary Table 2** (tissue samples).

### Cell Culture and Cell Transfection

The human breast cancer cell lines MCF-7, BT-549, normal mammary epithelial cell line MCF-10A, and human embryonic kidney cell line HEK293T were obtained from Cell Bank of Chinese Academy of Sciences (Shanghai, China). The cells were maintained in DMEM or RPMI-1640 medium supplemented with 10% (v/v) fetal bovine serum and 1% antibiotics at 37 °C in a 5% CO_2_ incubator.

The synthetic tiRNA single-strand mimics/inhibitor and corresponding negative control were transfected with Lipofectamine 2000 transfection reagent (Invitrogen). tRF-17-79MP9PP and the negative control (Ribobio, Guangzhou, China) were optimized for MCF-7 and BT-549 cells according to the manufacturer’s instructions. Short interfering (si)RNAs targeting THBS1 were obtained from GenePharma Co., Ltd (Shanghai, China). Three different siRNAs against THBS1 were chosen (si1855 sense strand: GCGUGAAGUGUACUAGCUATT, and anti-sense strand: UAGCUAGUACACUUCACGCTT; si2095 sense strand: CCAACAAACAGGUGUGCAATT, and anti-sense strand: UUGCACACCUGUUUGUUGGTT; si3461 sense strand: GCAUGACCCUCGUCACAUATT and anti-sense strand: UAUGUGACGAGGGUCAUGCTT). Cells were transiently transfected with individual siRNAs by using Lipofectamine 2000, then collected for subsequent assays after 48 h incubation.

### RNA Isolation, cDNA Synthesis and Quantitative PCR

Total RNAs were extracted from cells and tissues using Trizol reagent (Life Technologies, USA). While the total RNAs from serum of patients were isolated with Trizol LS reagent. And followed by removal of some RNA modifications with the rtStar™ tRF&tiRNA Pretreatment Kit (AS-FS-005, Arraystar, USA). Then, the RNA was quantified by RT-qPCR using a Bulge-Loop miRNA Stater Kit (Ribobio, China) following the manufacturer’s protocol and reverse transcribed to cDNA with Stem-loop RT primers specific for tRF-17-79MP9PP. RT reaction conditions of tRF-17-79MP9PP were 60 min at 42 °C and 10 min at 70 °C. Then qPCR was performed used the SYBR Green Mix containing Taq enzyme, dNTP mix, PCR buffer and SYBR Green I. RNU6B was used for tRF-17-79MP9PP template normalization. After adding forward primer and reverse primer, the mixtures were incubated at 95 °C for 10 min, followed by 40 cycles at 95 °C for 10 s, 60 °C for 20 s, and 70 °C for 10 s. RT-qPCR was performed as previously described (12). RT-qPCR for THBS1 and other genes was performed with SYBR^®^ Premix Dimer-Eraser (TaKaRa), β-actin was used for mRNA template normalization. The levels of tRF-17-79MP9PP and mRNA were calculated using 2^−ΔΔCt^ and 2^−ΔCt^ method for relative quantification of gene expression. The primers sequences were listed in **Supplementary Table 3**.

### Nuclear and Cytoplasmic RNA Fractionation

The isolations from cell nucleus and cytoplasm were obtained by the NE-PER Nuclear and Cytoplasmic Extraction Reagent (Thermo Fisher) following the manufacturer’s instructions. Briefly, a) 2 × 10^6^ cells were collected into 1.5 ml EP tube and centrifuged at 4 °C 500*g* for 5 min; b) remove the supernatant and add 200 μl of CER I pre-chilled on ice; c) shake violently for 15 s and then suspended for 10 min on ice; d) 11 μl CER II precooled on ice was added; e) shake violently for 5 s and stand on ice for 1 min; f) shake violently for 5 s, centrifuge at 4 °C 16,000*g* for 5 min; g) transfer the supernatant (cytoplasmic extract) to a pro-cooled EP tube and add 1 ml Magzol to extract cytoplasmic RNA; h) wash the pellet with 1 × PBS (nucleus extract), centrifuge at 4 °C 16,000*g* for 5 min; i) remove the supernatant, add 1 ml Magzol to lyse, for extracting nuclear RNA.

### Dual Luciferase Assay

A 60 nt length of the 3’UTR in THBS1 containing the predicted tRF-17-79MP9PP binding site (underlined) was ligated into pmirGLO-Reporter vector (GenePharma Co.) 3’ of the luciferase gene: 5’-CTCTGTTCTGCCTGGAAATTTAGGCTTCATACGGAAAGTGTTTGAGAGCAAGTAGTTGAC-3’ (THBS1 3’-UTR wild-type). While a negative control, the sequence of mutations was: 5’-CTCTGTTCTGCCTGGAAATTTAGGGA>GTATGCCTTTGTGTTTGAGAGCAAGTAGTTGAC-3’ (THBS1 3’-UTR mutant). The cells were co-transfected with wild-type or mutant reporter plasmid vector and tRF-17-79MP9PP mimics or inhibitor and corresponding negative control. After 48 h transfection, cells were harvested and analyzed by a Dual-luciferase reporter assay system (Promega).

### Western Blot Analysis

Total protein was extracted from cells and the protein concentration was determined using a BCA protein assay kit (Servicebio, #G2026). Equal amount of protein from each sample were separated by SDS-PAGE and transferred to polyvinylidene fluoride membranes, then incubated with primary antibodies directed against target proteins: THBS1 (#18304-1-AP; dilution 1:1,000; Proteintech); TGF-β1 (#21898-1-AP; dilution 1:1,000; Proteintech); Smad3 (#A11388; dilution 1:500; ABclonal); p-Smad3 (#AP0554; dilution 1:1,000; ABclonal); MMP3 (#GB11131; dilution 1:1,000; Servivebio); MMP9 (#GB12132-1; dilution 1:500; Servicebio); N-cadherin (#D119282-0100; dilution 1:1,000; BBI); ACTIN (#GB12001; dilution 1:1,000; Servicebio), and GAPDH (#KGAA002; dilution 1:1,000) chosen as a loading control.

### Cell Proliferation Assay

Cell viability and proliferation were detected using the Cell Counting Kit-8 (CCK-8; Dojindo, Laboratories, Japan). Cells were plated at a density of 3 × 10^3^ cells/well in 96-well plate. After 24, 48, and 72 h of incubation, the medium was replaced by 100 μl of fresh DMEM or RPMI-1640 and 10 μl CCK-8 reagent, then incubated for 2–3 h at 37 °C. Absorbance at 450 nm was determined by microplate reader. Each assay was repeated for three times.

### Colony Formation Assay

Cells were seeded onto 6-well plates at a density of 1 × 10^3^ cells per well and incubated for 7 days until visible clones appeared. Subsequently, cells were fixed with 4% paraformaldehyde and then stained with 1% crystal violet. The number of colonies was detected and counted under a light microscope.

### 5-Ethynyl-2’-Deoxyuridine (EdU) Incorporation Assay

Cells were incubated with Edu in medium (50 μM) for 2 h. Then, the cells were fixed in 4% paraformaldehyde for 30 min at room temperature, permeabilized with 0.5 TritoX-100, and stained with Apollo^®^ fluorescent dye for 1 h. Finally, DNA staining was performed with Hoechst33342 for 30 min, according to the manufacturer’s instruction of the Cell-Light Edu DNA cell proliferation kit from RiboBio. Photographs of the cells were scanned independently in a multi-tracking mode with an OLYMPUS confocal microscopy.

### Flow Cytometry Analysis of Cell Cycle

Cells were harvested and then washed with cold PBS (phosphate buffered saline) and fixed in 75% ethanol overnight at 4 °C and then incubated with RNase A for 20 min at 37 °C. Cells were subsequently stained with 400 μl propidium iodide solution for 30 min at room temperature in the dark. DNA content was then measured with FACSCalibur flow cytometer (BD Biosciences, USA). The percentage of the cells in G1, S, and G2 phase was analyzed. Each assay was repeated for three times.

### Transwell Assay

Briefly, the transfected cells were seeded into Transwell upper chamber (Coring Inc costar^®^, USA). The upper chamber was added with serum-free medium, and 500 μl medium containing 10% FBS in lower chamber. After 48 h incubation at 37 °C, the non-migrating cells remaining on the upper side of the filter were gently removed, and the cells on the lower surface of the inserts were fixed, stained and counted. As for cell invasion capability, the cells were seeded into Transwell upper chamber (Coring^®^ Matrigel^®^ invasion chamber, USA). The remaining steps were the same as the Transwell migration assay.

### Enzyme-Linked Immunosorbent Assay (ELISA)

After 48 h transfection, the supernatants of cells treated with tRF-17-79MP9PP mimics were collected, and TGF-β1 levels were detected with a Human/Mouse/Rat TGF-β1 ELISA Kit (EK981-48; MULTISCIENCES) according to the manufacturer’s instructions.

### Gene Ontology (GO) and Kyoto Encyclopedia of Genes and Genome (KEGG) Pathway Enrichment Analysis

For GO mapping, the GO terms for tRF-17-79MP9PP target genes based on homologies were extracted (http://www.geneontology.org). Integration Discovery (DAVID) software (http://david.abcC.ncifcrf.gov), was used to perform GO analysis to identify biological processes (BP), cellular components (CC), and molecular functions (MF) of these target genes. Meanwhile, KEGG pathways for target genes were retrieved from KEGG database (http://www.genome.jp/kegg/). GO or KEGG analyses with *P <*0.05 were considered as—statistically significant.

### Statistical Analysis

Data from biological triplicate experiments were presented as mean ± SEM. Student’s *t*-test was used for comparing two groups of data. The efficacy of tRF-17-79MP9PP was evaluated by sensitivity and specificity, the relationship of sensitivity-specificity was determined using Youden’s index. The Spearman correlation test was used to examine correlation between the expression of tRF-17-79MP9PP and 5’-tiRNA^Val^ in serum samples. All of the statistical testing results were conducted using SPSS 20.0 software and GraphPad Prism v8.0. A *P*-value of less than 0.05 was accepted as statistically significant.

## Results

### tRF-17-79MP9PP Is a Type of tRNA-Derived Fragment

**Figure 1:**
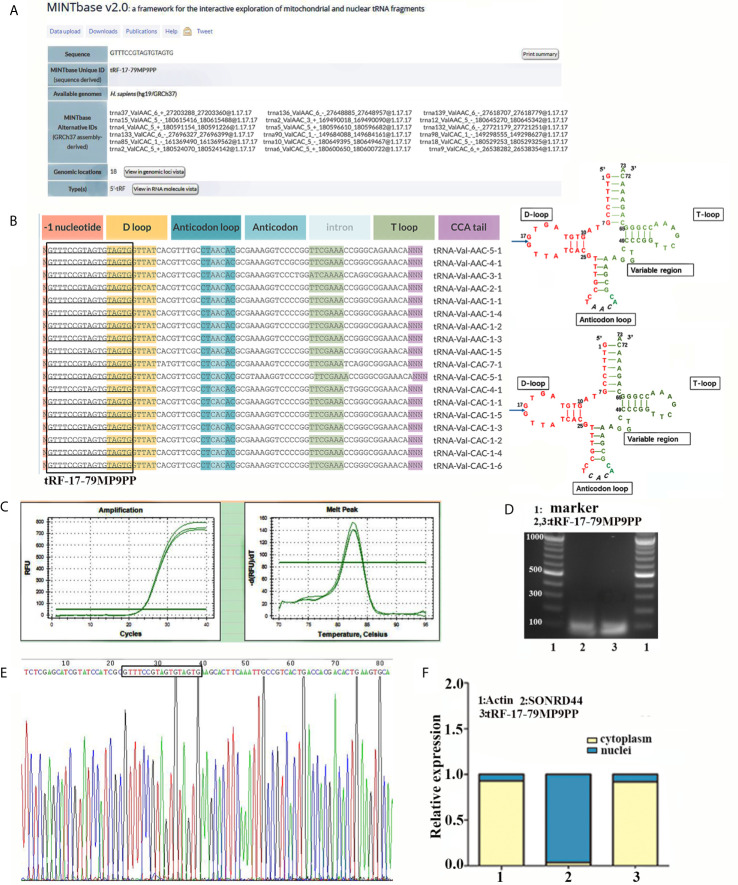
tRF-17-79MP9PP is a type of tRNA-derived fragment. **(A)** tRF-17-79MP9PP is a 5’- tRF fragment, with 18 genomic locations. **(B)** tRF-17-79MP9PP was derived from 5’- ends of mature tRNA-Val-AAC and tRNA-Val-CAC with the length of 17nt. The tRF-17-79MP9PP sequences are in the textbox and the arrow points to the cutting position. **(C)** The dissolution curve and amplification curve of tRF-17-79MP9PP. **(D)** The product of RT-PCR was run on 2% agarose gel. **(E)** The product of qRT-PCR was confirmed by Sanger sequencing. **(F)** Nucleocytoplasmic separation assay revealed that tRF-17-79MP9PP is mainly expressed in the cytoplasm. SONRD44 and actin were used as nuclear and cytoplasm localization markers, respectively.

In the MINTbase v2.0 (http://cm.jefferson.edu/MINTbase/), the molecule of tRF-17-79MP9PP belongs to a class of 17nt small RNAs and the sequence is 5’-GTTTCCGTAGTGTAGTG-3’. The fragments matched perfectly to the 5’ end of at least 18 annotated human tRNAs and the cleavage site is located on the D-loop in these sequences. The candidate mature tRNA carried the amino acid valine by the anticodon AAC or CAC (**Figures 1A, B** **)**. We detected the quantification of tRF-17-79MP9PP in breast cancer cells using qRT-PCR. **Figure 1C** displayed amplification curve and dissolution curve of tRF-17-79MP9PP. The product of qRT-PCR (10 μl) was run on 2% agarose gel electrophoresis, stained with EB (ethidium bromide), and visualized under UV illumination. The results showed a single electrophoresis band about 100 bp in size (**Figure 1D**). After recovering and cloning, we also performed the Sanger sequencing of the PCR product of qRT-PCR. The results showed that the sequence of tRF-17-79MP9PP is consistent with that of MINTbase v2.0, and the sequences matched perfectly (**Figure 1E**). Furthermore, the subcellular localization of tRF-17-79MP9PP was detected by quantifying nuclear/cytoplasmic RNA. The results revealed that tRF-17-79MP9PP transcriptions were more localized in the cytoplasm than in the nucleus (**Figure 1F**).

### Expression and Diagnosis Value of tRF-17-79MP9PP in Clinical Samples

**Figure 2:**
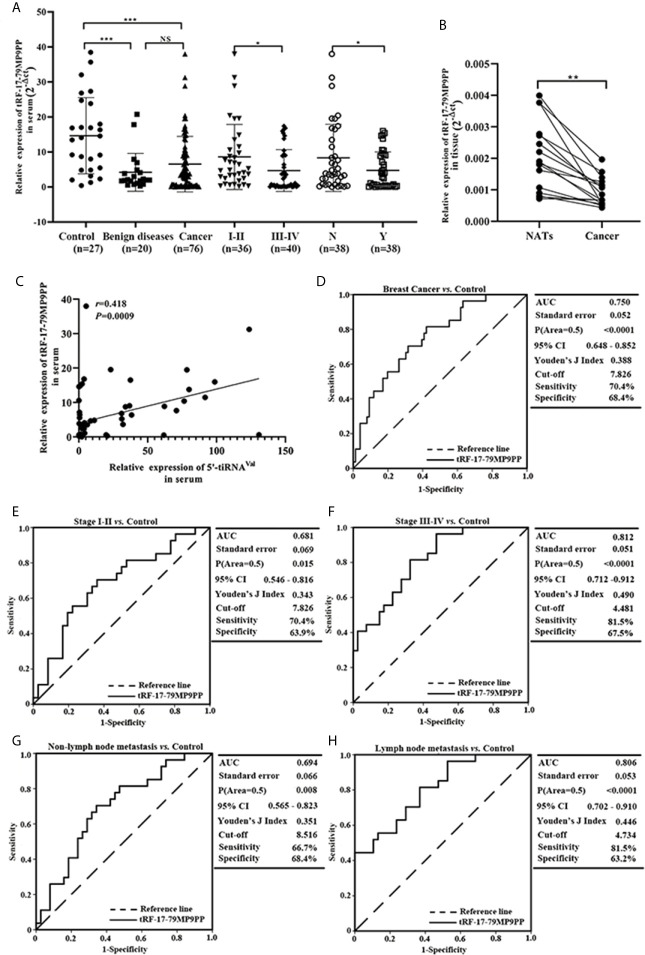
Expression and diagnostic value of tRF-17-79MP9PP in clinical sample. **(A)** Scatter plot representation of tRF-17-79MP9PP levels in serum from healthy controls, benign breast diseases patients and breast cancer patients. **(B)** Expression of tRF-17-79MP9PP levels was quantified by qRT-PCR in tissues and NATs. **(C)** The correlation between 5’-tiRNA^Val^ and tRF-17-79MP9PP in serum samples was analyzed with spearman correlation test. **(D)** The ROC-AUC of tRF-17-79MP9PP for differentiating all breast cancer patients from healthy control. **(E, F)** TNM stages I–II **(E)** and stages III–IV **(F)** in breast cancer from controls. **(G, H)** Non-Lymph node metastasis **(G)** and lymph node metastasis **(H)** in breast cancer from controls. U6 was used normalization. *p < 0.05; ** *p <* 0.01, *** *p <* 0.001 statistically significant. NATs, non-tumor adjacent tissues; ROC-AUC, Receiver Operating Characteristic-Area Under Curve; TNM, Tumor-Node-Metastasis. NS, no significance.

According to the previous research (15), we further detected the expression level of tRF-17-79MP9PP in serum from 76 patients and found that tRF-17-79MP9PP levels were significantly decreased in breast cancer patients and benign breast diseases patients, as compared to healthy controls (*p <*0.0001, **Figure 2A**). However, no statistically significant difference was found between cancer patients and benign breast diseases group (*p* = 0.0531). Moreover, lower expression of tRF-17-79MP9PP was observed in patients with higher TNM stages (stages I–II *vs.* stages III–IV: *p* = 0.0310) and lymph node metastasis (*p* = 0.0474). Meanwhile, we detected the expression level of tRF-17-79MP9PP in tissue from 16 pairs of breast cancer samples and found that tRF-17-79MP9PP level were significantly decreased in tumor tissues than NATs (*p* = 0.0002, **Figure 2B**). In our previous study, we found that another tRNA fragment, 5’-tiRNA^Val^ was also under-expressed in serum samples of breast cancer (12). Whether there is a correlation between these two tRNA fragments, although they are different types. The correlation between 5’-tiRNA^Val^ and tRF-17-79MP9PP in serum was analyzed with spearman correlation test. Intriguingly, a significant correlation was revealed (*r* = 0.418, *p* = 0.0009; **Figure 2C**). However, no significant correlation was observed between 5’-tiRNA^Val^ and tRF-17-79MP9PP in tissue (*p* = 0.2324). We preliminarily predicted that such results are probably from small amount of tissue samples. This result revealed that tRF-17-79MP9PP may be similar to 5’-tiRNA^Val^ in inhibiting breast cancer progression.

Furthermore, we evaluated the power of tRF-17-79MP9PP to discriminate the healthy controls and breast cancer groups with the area under the ROC curve (AUC). As shown in **Figure 2D** the ROC-AUC of tRF-17-79MP9PP for differentiating patients from healthy control was 0.750 (95% CI: 0.648–0.852), with a cut-off value of 7.826 (sensitivity: 70.4%, specificity: 68.4%). The ROC-AUC of 0.681 was specified for stages I–II from healthy control with 70.4% sensitivity and 63.9% specificity, and a cut-off value of 4.481 yielded a sensitivity of 81.5% and specificity of 67.5 in stages III–IV (**Figures 2E, F** **)**. While a ROC-AUC of 0.694 (95% CI, 0.565–0.823) differentiated non-lymph node metastasis of patients from control, with a sensitivity of 66.7% and a specificity of 68.4%. The sensitivity and specificity of differentiated lymph node metastasis of patients from control was 81.5 and 63.2%, respectively (**Figures 2G, H** **)**. The above results demonstrated that tRF-17-79MP9PP levels may be as a potential diagnostic marker for breast cancer.

### tRF-17-79MP9PP Suppresses Breast Cancer Cell Proliferation, Migration and Invasion

**Figure 3:**
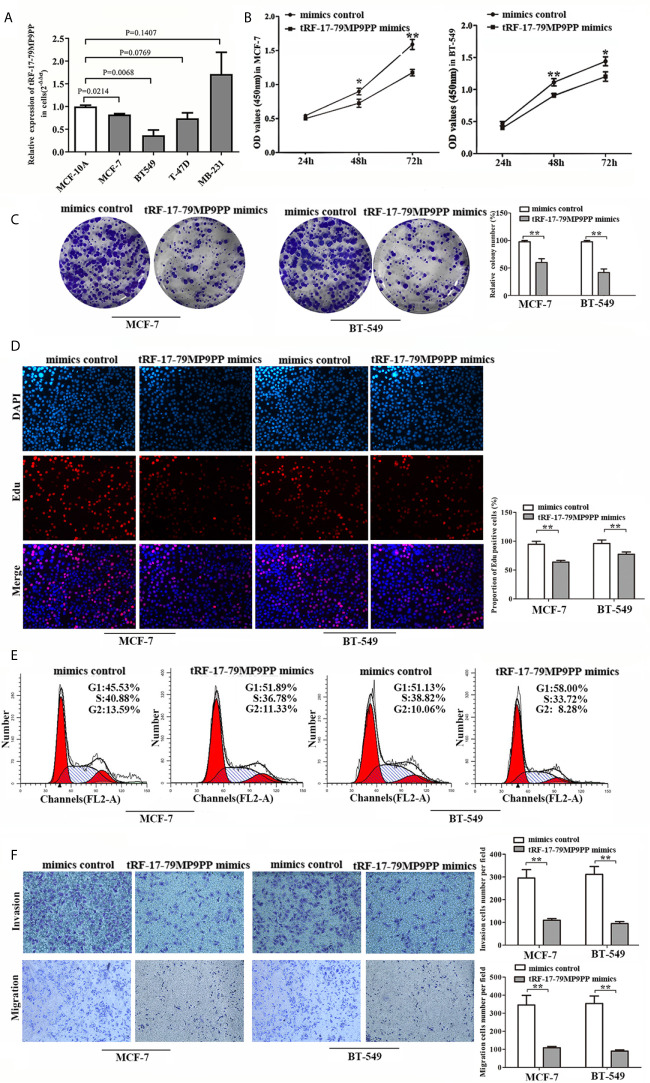
tRF-17-79MP9PP suppresses breast cancer cell malignant activity. **(A)** The expression of tRF-17-79MP9PP in normal mammary epithelial cell lines MCF-10A and breast cancer cells. **(B)** Cell viability of breast cancer cell lines after transfection with tRF-17-79MP9PP mimics or mimics control and detection by CCK-8 assay. **(C, D)** Colony formation assay and Edu assay of treated cells. **(E)** Cell cycle analysis in treated cells by flow cytometry. **(F)** Representative images and bar graphs were depicted to investigate the migration and invasion ability in treated cells. *p < 0.05; ** *p <* 0.01, statistically significant.

Next, we detected the expression levels of tRF-17-79MP9PP in breast cancer cells, MCF-7, BT549, T-47D and MDA-MB-231. qRT-PCR analysis showed that tRF-17-79MP9PP levels were significantly lower in MCF-7 and BT549 cells than in MCF-10A cells (**Figure 3A**). Further investigation the biological roles of tRF-17-79MP9PP in breast cancer, we transfected the tRF-17-79MP9PP mimics and control RNA into MCF-7 and BT549 cells. The CCK-8 assay and colony formation assay demonstrated that overexpression of tRF-17-79MP9PP significantly decreased the growth rate and colony formation of MCF-7 and BT549 cells (**Figures 3B, C** **)**. The level of DNA synthesis, as examined using the EdU incorporation assay, was significantly reduced in tRF-17-79MP9PP transfected cells compared to control cells (**Figure 3D**). Moreover, cell cycle analysis showed that overexpression of tRF-17-79MP9PP significantly increased the percentage of cells in the G1 peak while decreasing the percentage of cells in the S phase (**Figure 3E**). Meanwhile, we also found that tRF-17-79MP9PP overexpression markedly reduced the migration and invasion of breast cancer cells (**Figure 3F**).

### GO and KEGG Enrichment Analysis of tRF-17-79MP9PP Target Genes

**Figure 4:**
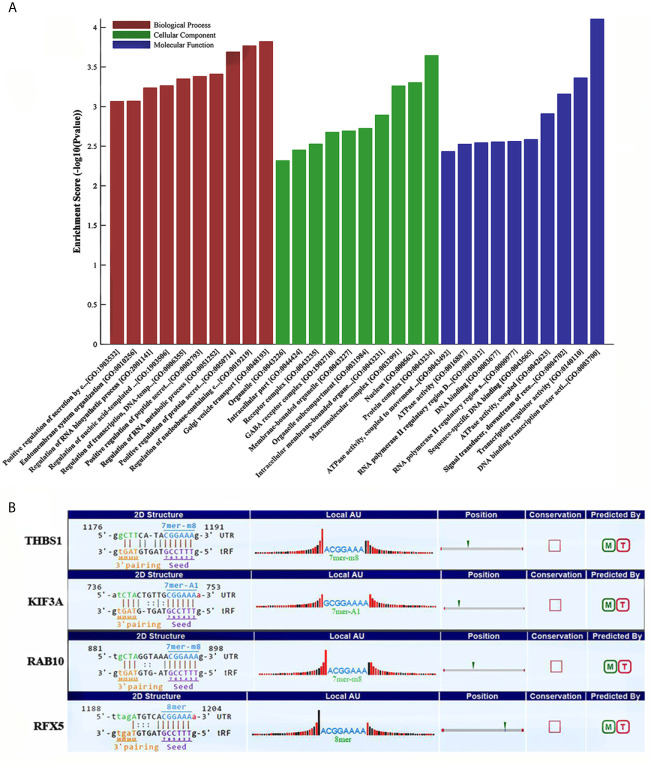
GO and KEGG enrichment analysis of tRF-17-79MP9PP target genes. **(A)** GO analysis of the DEGs-tRF-17-79MP9PP target genes enriched in biological process (BP), Cellular component (CC) and Molecular function (MF). **(B)** The 3’UTR area of the four candidate genes (*THBS1*, *KIF3A*, *RAB10*, *RFX5*) combined with tRF-17-79MP9PP.

Some studies have demonstrated that tRFs with a similar function to miRNAs can sequence-specifically silence the mRNA expression (17). We therefore have analyzed potential target genes of tRF-17-79MP9PP with GO and KEGG enrichment analysis to explore the molecular mechanism of tRF-17-79MP9PP in breast cancer. The results of GO analysis of the DEGs-tRF-17-79MP9PP target genes are shown in **Figure 4A**, which revealed that these target genes were significantly enriched in biological processes (BP) of regulation of protein secretion, regulation of RNA metabolic process, RNA biosynthetic process and endomembrane system organization. The significant cellular component (CC) terms included protein complex, nucleus, macromolecular complex, membrane-bounded organelle and intracellular part. Molecular function (MF) terms included DNA binding transcription factor activity, transcription regulator activity, signal transducer, downstream of receptor, with serine/threonine kinase activity, sequence-specific DNA binding and DNA binding.

Enriched signaling pathways for target genes of tRF-17-79MP9PP identified by KEGG pathway analysis were ranked according to the *P*-values (**Table 1**). In total, 103 host genes had KEGG pathway annotations. The significant 19 signaling pathways included AMPK signaling pathway, ECM-receptor interaction, focal adhesion, antigen processing and presentation, TGF-beta signaling pathway, platelet activation and so on.

**Table 1:** KEGG pathway analysis for predicted target genes of tRF-17-79MP9PP.

ID	Definition	Selection counts	*P*-value	Genes
**hsa04152**	AMPK signaling pathway	8	0.000316972	CAMKK2//CREB3L2//GYS2//IGF1R//MAP3K7//PIK3CD//RAB10//SCD
**has05033**	Nicotine addiction	4	0.002505722	GABRD//GABRR3//GRIN3A//SLC17A8
**hsa04550**	Signaling pathways regulating pluripotency of stem cells	7	0.003820292	BMI1//BMPR1A//BMPR2//COMMD3-BMI1//IGF1R//JARID2//PIK3CD
**hsa04670**	Leukocyte transendothelial migration	6	0.005432806	ARHGAP35//EZR//ITGA4//JAM3//PIK3CD//PLCG2
**hsa04512**	ECM-receptor interaction	5	0.006428242	COL4A1//COL6A5//ITGA4//ITGA6//THBS1
**hsa04510**	Focal adhesion	8	0.007841788	ATG16L1//CAMKK2//IGF1R//MAP3K7//PIK3CD//STX17
**hsa04140**	Autophagy - animal	6	0.01024463	
**hsa04130**	SNARE interactions in vesicular transport	3	0.01261484	STX17//STX2//VTI1A
**hsa04933**	AGE-RAGE signaling pathway in diabetic complications	5	0.01391275	COL4A1//NFATC1//PIK3CD//PLCG2//PRKCZ
**hsa04066**	HIF-1 signaling pathway	5	0.01448136	IGF1R//MKNK1//PDHB//PIK3CD//PLCG2
**hsa05340**	Primary immunodeficiency	3	0.01587893	DCLRE1C//RFX5//TAP1
**hsa04668**	TNF signaling pathway	5	0.01960006	CASP10//CREB3L2//DNM1L//MAP3K7//PIK3CD
**hsa04612**	Antigen processing and presentation	4	0.0247631	HLA-DPA1//NFYA//RFX5//TAP1
**hsa04151**	PI3K-Akt signaling pathway	10	0.02949896	COL4A1//COL6A5//CREB3L2//GYS2//IGF1R//ITGA4//ITGA6//PIK3CD//PRLR//THBS1
**hsa04611**	Platelet activation	5	0.03212829	ARHGAP35//FGB//PIK3CD//PLCG2//PRKCZ
**hsa04350**	TGF-beta signaling pathway	4	0.03273202	BMP6//BMPR1A//BMPR2//THBS1
**hsa04380**	Osteoclast differentiation	5	0.03720821	LILRA6//MAP3K7//NFATC1//PIK3CD//PLCG2
**hsa04211**	Longevity regulating pathway	4	0.03924477	CAMKK2//CREB3L2//IGF1R//PIK3CD
**hsa04360**	Axon guidance	6	0.04039088	BMPR2//PIK3CD//PLCG2//PRKCZ//SEMA7A//UNC5D

Subsequently, we found that a common pathway identified with GO and KEGG enrichment analysis was antigen processing and presentation (GO ID: 0019882; Pathway ID: hsa04612), which decrease the scope in gene selection. Among them, the genes contained in GO 0019882 were *TAP1*, *PSMA8*, *PSMD9*, *THBS1*, *KIF3A*, *HLA-DPA1*, *RAB10*; Pathway hsa04612 contained the following genes: *HLA-DPA1*, *NFYA*, *RFX5*, *TAP1*. The above experimental data confirmed that tRF-17-79MP9PP could inhibit breast malignant activities of cancer cells, so its target gene should be associated with tumor cell invasion, metastasis or proliferation. Based on the rationale, four candidate genes (*THBS1*, *KIF3A*, *RAB10*, *RFX5*) were selected after a literature review (18–23). Further analysis revealed that tRF-17-79MP9PP had “seed sequences”, which may bind to the 3’UTR of the four candidate genes according to TargetScan and miRanda programs (**Figure 4B**).

### tRF-17-79MP9PP Reduces THBS1 Expression by Directly Targeting Its 3’UTR

**Figure 5:**
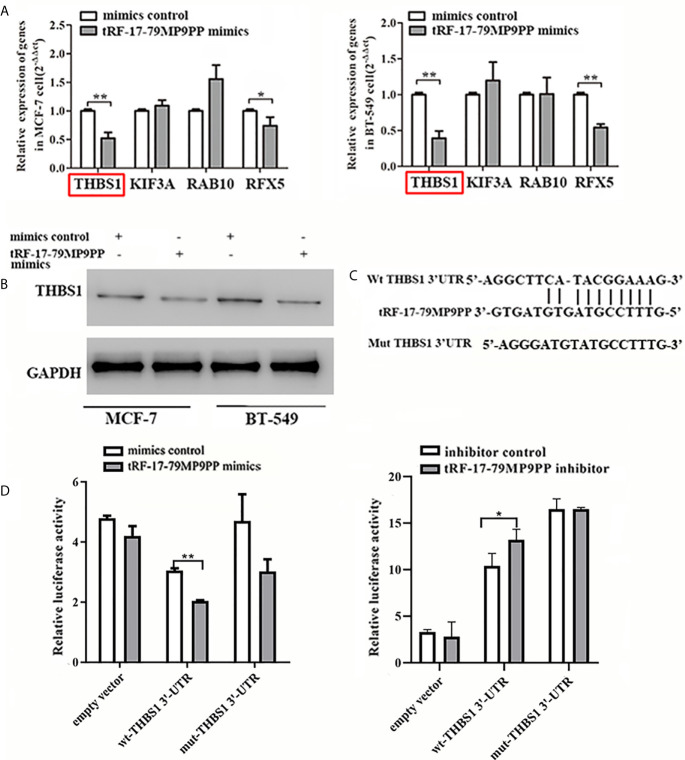
tRF-17-79MP9PP directly regulates THBS1 expression in breast cancer cells. THBS1 mRNA **(A)** and protein **(B)** levels expression in treated MCF-7 and BT549 cells, respectively. **(C)** 3’-UTR fragment of wide-type (wt) and mutated which disrupted interaction with tRF-17-79MP9PP. **(D)** The wt or mutated reporter plasmid was co-transfected with tRF-17-79MP9PP mimics/inhibitor and negative control into HEK293T cells. Luciferase activity of wt THBS1 3’-UTR was significantly influenced by tRF-17-79MP9PP in cells. *p < 0.05; ** *p <* 0.01, statistically significant.

The expression of four candidate genes was further validated using qRT-PCR. THBS1 mRNA levels were significantly downregulated by tRF-17-79MP9PP in MCF-7 cells and BT549 cells compared with the negative controls (**Figure 5A**). In addition, overexpression of tRF-17-79MP9PP induced a significant decrease of THBS1 protein levels in cells (**Figure 5B**). These results suggest that THBS1 expression is down-regulated by tRF-17-79MP9PP in breast cancer cells.

In order to further explore the regulation of THBS1 by tRF-17-79MP9PP, we constructed vectors expressing of 3’UTR of *THBS1* (**Figure 5C**). The wild type or mutant constructs were co-transfected with tRF-17-79MP9PP mimics/inhibitor and negative control into HEK293T cells. After 48 h transfection, the related luciferase activity in HEK293T transfected with wild type reporter plasmid was significantly reduced by the mimics of tRF-17-79MP9PP compared to control RNA, while the cells transfected with inhibitor exhibited dramatical enhancement of luciferase activity. However, tRF-17-79MP9PP mimics and inhibitor had no effect on the luciferase activity in the mutant reporter plasmid transfected cells (**Figure 5D**). Taken together, these results indicated that tRF-17-79MP9PP might directly target THBS1 and regulate its expression.

### Attenuation of THBS1 Expression Rescues the tRF-17-79MP9PP-Mediated Inhibitory Effects on Breast Cancer Cells

**Figure 6:**
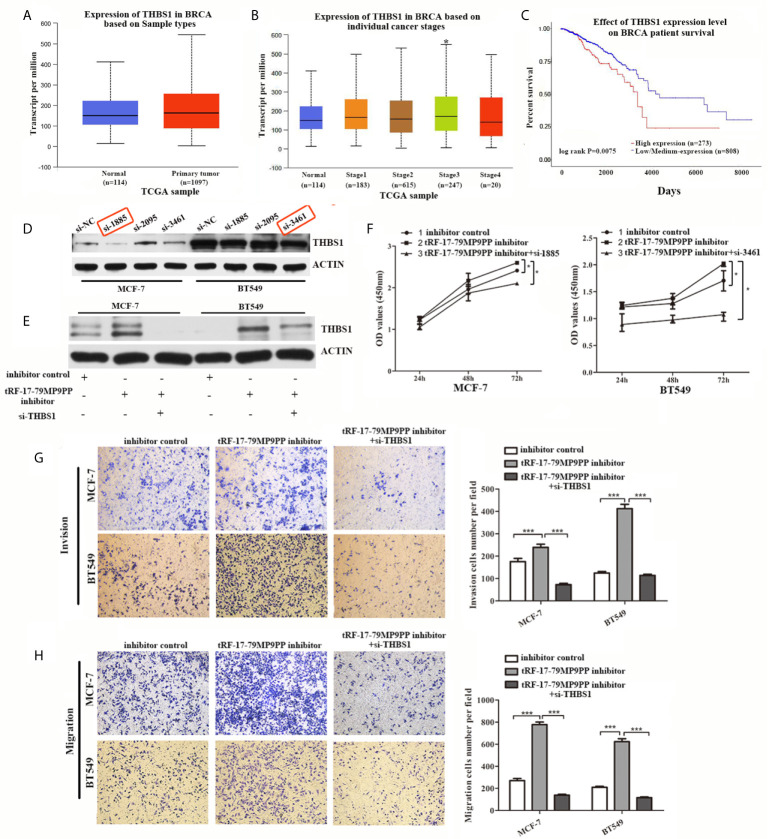
Attenuation of THBS1 expression rescues the tRF-17-79MP9PP-mediated inhibitory effects on breast cancer cells. **(A)** The expression of THBS1 between cancer and normal samples in TCGA database. **(B)** Expression of THBS1 in breast cancer based on individual cancer stages in TCGA database. **(C)** Prognostic effect of THBS1 for breast cancer was displayed in TCGA database. **(D)** Protein levels in MCF-7 and BT549 cells transfected with THBS1 siRNA or siRNA negative control. **(E)** Western-blot analysis was used to detect THBS1 expression in cells transfected with tRF-17-79MP9PP inhibitor, si-THBS1 or negative control. **(F)** Cell viability of MCF-7 and BT549 cells after transfected with tRF-17-79MP9PP inhibitor, si-THBS1 or negative control was detected by CCK-8 assay. Cell Migration assays **(G)** and invasion assays **(H)** were performed in treated cells. *p < 0.05; *** *p <* 0.001 statistically significant.

THBS1 was the first member to be identified in the thrombospondins family and is a main player in the tumor microenvironment (24). However, the role of THBS1 in breast cancer development has not been characterized. First at all, we analyzed the expression and prognostic value of THBS1 in public databases: TCGA (The Cancer Genome Atlas;http://ualcan.path.uab.edu). Statistical analysis in TCGA database revealed that no significant difference of THBS1 expression was observed between cancer and normal samples (**Figure 6A**). Nevertheless, when patients were segregated based upon cancer TNM stage, the levels of THBS1 in stage III was significantly higher than normal samples in TCGA breast invasive carcinoma database (*p* = 0.041; **Figure 6B**). Kaplan–Meier survival analysis indicated that the overall survival in breast cancer patients with high THBS1 expression was significantly reduced in TCGA breast invasive carcinoma database (*p* = 0.0075, **Figure 6C**).

To determine whether the dysregulation of THBS1 is involved in the regulation of cell proliferation, migration and invasion by tRF-17-79MP9PP, we used specific siRNAs against THBS1 to knock down THBS1 expression and verified that si-1855 and si-3461 have relatively high knockdown efficiency for both MCF-7 and BT549, respectively (**Figure 6D**). These two siRNAs were used in the following research. As shown in **Figure 6E**, co-transfected with THBS1 siRNA markedly abolished the elevated expression of THBS1 induced by the tRF-17-79MP9PP inhibitor in breast cancer cells by a “rescue” experiment. Furthermore, reduction of THBS1 expression also partially recovered the effects of tRF-17-79MP9PP inhibition on breast cancer cell viability, invasion and migration compared to that in the control group (**Figures 6F–H** ****). These data revealed that tRF-17-79MP9PP inhibits breast cancer cell malignant activities in a THBS1-mediated manner.

### tRF-17-79MP9PP Expression Suppresses the TGF-β1/Smad3 Pathway Through Targeting THBS1

**Figure 7:**
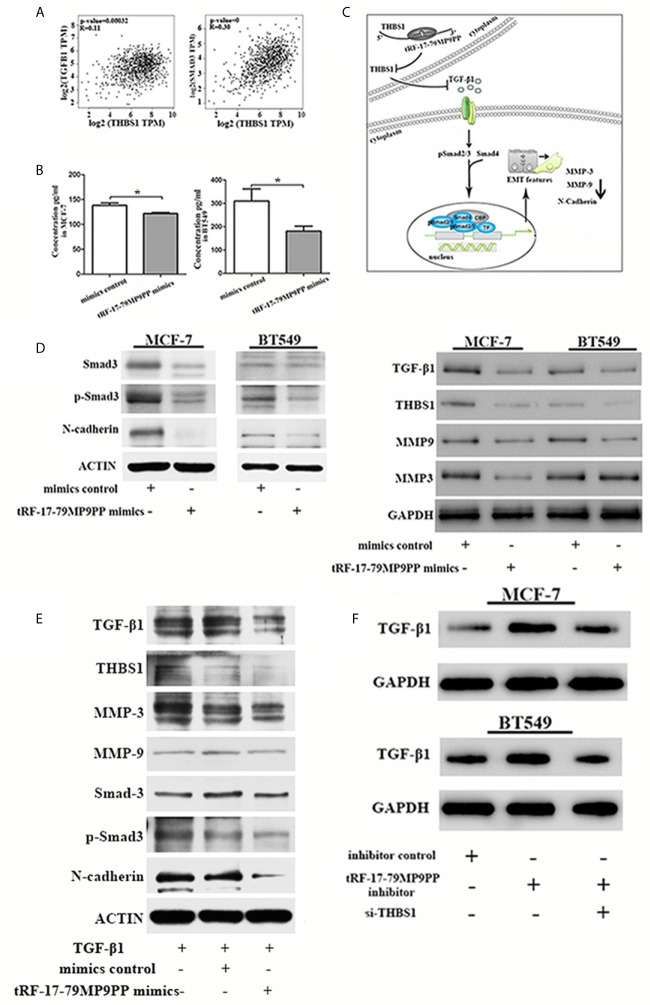
tRF-17-79MP9PP regulates TGF-β1 signaling pathway. **(A)** The expression correlation between THBS1 and TGF-β1 and Smad3 genes in clinical breast cancer specimens. **(B)** The amounts of TGF-β1 in the culture supernatants of MCF-7 and BT549 cells determined by ELISA. Data represent three independent experiments. **(C)** Proposed working model. tRF-17-79MP9PP-mediated THBS1 inhibition may lead to the inactivation of TGF-β1/Smad3 signaling pathway in breast cancer cells. **(D)** Western-blot analysis protein levels in breast cancer cells transfected with tRF-17-79MP9PP mimics or control. **(E)** Western-blot analysis protein levels in transfected cells with TGF-β1 treatment in MCF-7 cell. **(F)** Western-blot analysis protein levels in MCF-7 and BT549 cells co-transfected with tRF-17-79MP9PP inhibitor and si-THBS1. β-actin or GAPDH was used as a loading control. *p < 0.05 statistically significant.

Epithelial-to-mesenchymal transition (EMT) is regulated by multiple signaling pathways including TGFβ, AKT, Notch and Wnt (25–27). Some experiments suggest that the TGF-β1 mediated stimulation of THBS1 expression is a common mechanism governing many types of epithelial and mesenchymal cells in the tumor microenvironment (28). Subsequently, to validate the expression correlation between THBS1 and TGF-β1, and their related genes in clinical breast cancer specimens, we used TCGA data to perform co-expression analyses *via* GEPIA (Gene Expression Profiling Interactive Analysis; http://gepia.cancer-pku.cn/index.html). As shown in **Figure 7A**, THBS1 expression was positively associated with TGF-β1 (*r* = 0.11, *p <*0.001) and Smad3 (*r* = 0.39, *p <*0.001) in breast cancer. The ELISA results also demonstrated that the level of TGF-β1 in the supernatants of tRF-17-79MP9PP mimics transfected cells was lower than that in control cells, indicating that tRF-17-79MP9PP inhibited the secretion of TGF-β1(**Figure 7B**).

Therefore, we hypothesized that tRF-17-79MP9PP-mediated THBS1 inhibition may lead to the inactivation of TGF-β1/Smad3 signaling pathway in breast cancer cells (**Figure 7C**). We detected the protein expression of THBS1, TGF-β1, Smad3, phosphorylation of smad3 (p-Smad3) and other protein related to EMT in breast cancer cells transfected with tRF-17-79MP9PP mimics or controls. The western blot assay showed that the expression levels of Smad3, p-Smad3, TGF-β1, THBS1, MMP-9, MMP-3 and N-cadherin protein were decreased by the tRF-17-79MP9PP mimics (**Figure 7D**). And then, we treated transfected tRF-17-79MP9PP mimics cells with 5ng/ml Recombined Human TGF-β1(PeproTech, USA) for 24 h, and analyzed the downstream protein expression. As shown in **Figure 7E**, TGF-β1 caused an increase in Smad3, p-Smad3, MMP3, MMP-9 and N-cadherin expression, but the effect was partially reversed by adding tRF-17-79MP9PP mimics in MCF-7 cells. Unexpectedly, the inhibitory effect of tRF-17-79MP9PP mimics on THBS1 was not obvious after TGF-β1 treatment.

To further verify the influence of tRF-17-79MP9PP on regulation of TGF-β1/Smad3 signaling pathway through THBS1, tRF-17-79MP9PP inhibitor and si-THBS1 were co-transfected into breast cancer cells. In the **Figure 7F** we found that THBS1 silence partially reversed the inhibition effect of tRF-17-79MP9PP on TGF-β1 protein expression in cells. Taken together, these data suggested that tRF-17-79MP9PP can suppress the TGF-β1/Smad3 signaling pathway by targeting THBS1 in breast cancer cells.

## Discussion

Breast cancer is still the leading cause of cancer-related deaths in worldwide women due to late diagnosis, lack of treatment options and cancer heterogeneity. Although decades of research have provided considerable insight into the multistep metastatic process, the molecular basis of breast cancer development process remains largely unknown (29). With the development of sequencing technology, new types of small non-coding RNAs including tRNAs and their derivatives have been identified in different cancer types (11, 30, 31), including ovarian cancer (32), gastric cancer (33) and colorectal cancer (34). As our group has previously reported tRF&tiRNA in breast cancer (15), one of the tRFs, tRF-17-79MP9PP might affect breast cancer as well. tRF-17-79MP9PP is a 5’-tRF fragment and mainly located in the cytoplasm. Here we demonstrated that tRF-17-79MP9PP was markedly downregulated in breast cancer cells, tissue and serum samples, and showed a negative correlation with cancer TNM stage and lymph node metastasis. Meanwhile, the ROC-AUC for tRF-17-79MP9PP was 0.750 (95% CI, 0.648–0.852) for differentiating all breast cancer patients from healthy controls with 70.4% sensitivity and 68.4% specificity at a cut-off value of 7.826. Because of the limited number of cases, the diagnostic efficacy of tRF-17-79MP9PP is not ideal. Next, we will increase the number of research subjects to further evaluate the diagnostic efficacy and therapeutic effect of tRF-17-79MP9PP for breast cancer. In addition, we detected that overexpression of tRF-17-79MP9PP significantly suppresses the ability of breast cancer cells proliferation, migration and invasion. The above results revealed that tRF-17-79MP9PP may act as an inhibitor in breast cancer.

tRFs, which paly a similar role with miRNA by silencing a series of target gene expression, acting in a similar way by Argonaut proteins interaction and involving Dicer in their generation (30, 31). Some tRFs are known to directly bind mRNA target in manner similar to canonical miRNAs, such as APOER2 as an endogenous target of tRF5-GluCTC in respiratory tract infection caused by respiratory syncytial virus (35), the generation of tRF-CU1276 is dependent on Dicer1, which can be associated with Argonaut proteins and inhibit proliferation and modulates the molecular response to DNA damage by silencing expression of its target gene PRA1 in B cell lymphoma (36). In this study, we found that tRF-17-79MP9PP downregulated its target gene THBS1. Immediately after, luciferase assay was used to verify the target site of tRF-17-79MP9PP, which demonstrated that the predicted site in the 3’-UTR of THBS1 was capable of binding tRF-17-79MP9PP.

THBS1 has been identified as a protein that is released by activated platelets, which is factors sharing pro-fibrotic and anti-angiogenic properties (28, 37). This gene was considered the most effective pro-fibrotic factor involved in the development of myelofibrosis and other fibrotic states (38, 39). Some studies suggest that THBS1 to be a biomarker for monitoring a primary myelofibrosis targeted therapy (37). Moreover, THBS1 is a secreted protein that acts in the tumor microenvironment to inhibit angiogenesis, regulate antitumor immunity, tumor cell migration, and influence the activities of extracellular proteases and growth factor, which plays an important role in tumor progression (18, 40). In our study, we also found that the levels of THBS1 in advance stage were significantly higher than normal samples in TCGA breast invasive carcinoma database. Kaplan–Meier survival analysis demonstrated that the overall survival of breast cancer patients with high THBS1 expression was significantly decreased in TCGA breast invasive carcinoma database. Importantly, rescue experiments and functional assays displayed that the suppression of THBS1 partially abolished the inhibitory effect of tRF-17-79MP9PP on proliferation and metastasis of MCF-7 and BT549 cells.

THBS1 is also identified as a major physiological activator of TGF-β, a potent elicitor of EMT (41). A previous study showed that THBS1 is the chief tumor-specific ECM protein that froms the tumor microenvironment collaboratively with TGF-β1 to favor oral squamous cell carcinoma invasion (28). However, it remains unclear whether THBS1 participates in breast cancer. Shen et al. once reported a new signal axis, YAP/THBS1/FAK, in the modulation of adhesion and invasiveness of breast cancer (42). In the present study, we showed that THBS1 expression was positively associated with TGF-β1 and Smad3 in clinical breast cancer specimens by use of public database. Therefore, we hypothesized that tRF-17-79MP9PP might contribute to breast cancer progression *via* the THBS1/TGF-β1/Smad3 pathway. Our data suggested that tRF-17-79MP9PP inhibits the THBS1-mediated TGF-β1/Smad3 pathway and the EMT related genes (MMP3, MMP-9 and N-cadherin) in breast cancer cells. Stimulation of THBS1 expression by TGF-β1 was observed in oral squamous cell carcinoma, lung carcinoma cells A549, skin keratinocytes HaCaT, and rat proximal tubular epithelial cells NRK52E (28, 43–45). And then, we treated transfected tRF-17-79MP9PP mimics cells with TGF-β1, and analyzed THBS1 expression and downstream protein expression. Interestingly, tRF-17-79MP9PP mimics partially attenuated TGF-β1-induced Smad3, p-Smad3 and EMT related protein expression. Unfortunately, the effect was not obvious for THBS1. A partial explanation for the result might be exogenous TGF-β1 consumed tRF-17-79MP9PP mimics and weakened its effect on THBS1. Another possible reason is that the stimulation of THBS1 by TGF-β1 was stronger than the repression of THBS1 by tRF-17-79MP9PP mimics, so that THBS1 expression level in mimics group is similar to that of the control group. We will also further explore other possible reasons. Even though, we found that reduction of THBS1 expression partially reversed the inhibition effect of tRF-17-79MP9PP on TGF-β1 protein expression in cells in the present study, as well as in the previous study showing the addition of THBS1 antibody, αvβ3 and CD47 reduced TGF-β1 activation in the MCF-7 breast cancer cells treated with tamoxifen (46). The results suggest that tRF-17-79MP9PP may regulate TGF-β1 through THBS1 in breast cancer cells.

In sum, we confirmed that tRF-17-79MP9PP significantly suppresses cells malignant activities as a new tumor-suppressor through the THBS1/TGF-β1/Smad3 axis in breast cancer. The study might provide a potential diagnostic and therapeutic target for breast cancer.

## Data Availability Statement

The original contributions presented in the study are included in the article/**Supplementary Material**. Further inquiries can be directed to the corresponding author.

## Ethics Statement

The studies involving human participants were reviewed and approved by the Ethics Committee of Nanjing Medical University. The patients/participants provided their written informed consent to participate in this study.

## Author Contributions

Conception and design: DM and FY. Provision of study materials or patients: FH, HC, and JZ. Collection and assembly of data: FH, HC, and JZ. Data analysis and interpretation: DM, LT, and FY. Manuscript writing: DM. Final approval of manuscript: All authors. All authors contributed to the article and approved the submitted version.

## Funding

This study was supported by the National Natural Science Foundation of China (NO. 81871718), the National Natural Science Foundation of China (NO. 82002225), and the Natural Science Foundation of Jiangsu Province (BK20181090).

## Conflict of Interest

The authors declare that the research was conducted in the absence of any commercial or financial relationships that could be construed as a potential conflict of interest.

## Abbreviations

THBS1, Thrombospondin-1; TGF-β, Transformation growth factor; EMT, Epithelial-to-mesenchymal transition; ECM, Extracellular matrix; DMEM, Dulbecco’s modified Eagle’s medium; TNM, Tumor-Node-Metastasis; qRT-PCR, Quantitative real time-PCR; ROC-AUC, Receiver Operating Characteristic-Area Under Curve.

## References

[1] Ghildiyal M Zamore PD. Small silencing RNAs: an expanding universe. Nat Rev Genet (2009) 10(2):94–108. 10.1038/nrg2504 19148191 PMC2724769

[2] Pompon J Garcia-Blanco MA. RNA: jack of all trades and master of all. Cell (2015) 160(4):579–80. 10.1016/j.cell.2015.01.047 25679756

[3] Esteller M. Non-coding RNAs in human disease. Nat Rev Genet (2011) 12(12):861–74. 10.1038/nrg3074 22094949

[4] Cech TR Steitz JA. The noncoding RNA revolution-trashing old rules to forge new ones. Cell (2014) 157(1):77–94. 10.1016/j.cell.2014.03.008 24679528

[5] Cole C Sobala A Lu C Thatcher SR Bowman A Brown JW. Filtering of deep sequencing data reveals the existence of abundant Dicer-dependent small RNAs derived from tRNAs. Rna (2009) 15(12):2147–60. 10.1261/rna.1738409 PMC2779667 19850906

[6] Lee YS Shibata Y Malhotra A Dutta A. A novel class of small RNAs: tRNA-derived RNA fragments (tRFs). Genes Dev (2009) 23(22):2639–49. 10.1101/gad.1837609 PMC2779758 19933153

[7] Elkordy A Mishima E Niizuma K Akiyama Y Fujimura M Tominaga T. Stress-induced tRNA cleavage and tiRNA generation in rat neuronal PC12 cells. J Neurochem (2018) 46(5):560–9. 10.1111/jnc.14321 29431851

[8] Shigematsu M Kirino Y. 5’-Terminal nucleotide variations in human cytoplasmic tRNAHisGUG and its 5’-halves. Rna (2017) 23(2):161–8. 10.1261/rna.058024.116 PMC5238791 27879434

[9] Keam SP Hutvagner G. tRNA-Derived Fragments (tRFs): Emerging New Roles for an Ancient RNA in the Regulation of Gene Expression. Life (2015) 5(4):1638–51. 10.3390/life5041638 PMC4695841 26703738

[10] Shen Y Yu X Zhu L Li T Yan Z Guo J. Transfer RNA-derived fragments and tRNA halves: biogenesis, biological functions and their roles in diseases. J Mol Med (2018) 96(11):1167–76. 10.1007/s00109-018-1693-y 30232504

[11] Zhu L Ge J Li T Shen Y Guo J. tRNA-derived fragments and tRNA halves: The new players in cancers. Cancer Lett (2019) 452:31–7. 10.1016/j.canlet.2019.03.012 30905816

[12] Mo D Jiang P Yang Y Mao X Tan X Tang X. A tRNA fragment, 5’-tiRNA(Val), suppresses the Wnt/beta-catenin signaling pathway by targeting FZD3 in breast cancer. Cancer Lett (2019) 457:60–73. 10.1016/j.canlet.2019.05.007 31078732

[13] Zhang M Li F Wang J He W Li Y Li H. tRNA-derived fragment tRF-03357 promotes cell proliferation, migration and invasion in high-grade serous ovarian cancer. OncoTargets Ther (2019) 12:6371–83. 10.2147/OTT.S206861 PMC6702494 31496739

[14] Weigelt B Peterse JL van ‘t Veer LJ. Breast cancer metastasis: markers and models. Nat Rev Cancer (2005) 5(8):591–602. 10.1038/nrc1670 16056258

[15] Wang X Yang Y Tan X Mao X Wei D Yao Y. Identification of tRNA-Derived Fragments Expression Profile in Breast Cancer Tissues. Curr Genomics (2019) 20(3):199–213. 10.2174/1389202920666190326145459 31929727 PMC6935952

[16] Sinn HP Kreipe H. A Brief Overview of the WHO Classification of Breast Tumors, 4th Edition, Focusing on Issues and Updates from the 3rd Edition. Breast Care (2013) 8(2):149–54. 10.1159/000350774 PMC3683948 24415964

[17] Li S Xu Z Sheng J. tRNA-Derived Small RNA: A Novel Regulatory Small Non-Coding RNA. Genes (2018) 9(5):246. 10.3390/genes9050246 PMC5977186 29748504

[18] Liu X Xu D Liu Z Li Y Zhang C Gong Y. THBS1 facilitates colorectal liver metastasis through enhancing epithelial-mesenchymal transition. Clin Trans Oncol Off Publ Fed Spanish Oncol Soc Natl Cancer Inst Mexico (2020) 22(10):1730–40. 10.1007/s12094-020-02308-8 32052380

[19] Wang W Zhang R Wang X Wang N Zhao J Wei Z. Suppression of KIF3A inhibits triple negative breast cancer growth and metastasis by repressing Rb-E2F signaling and epithelial-mesenchymal transition. Cancer Sci (2020) 111(4):1422–34. 10.1111/cas.14324 PMC7156822 32011034

[20] Li YF Pei FL Cao MZ. CircRNA_101951 promotes migration and invasion of colorectal cancer cells by regulating the KIF3A-mediated EMT pathway. Exp Ther Med (2020) 19(5):3355–61. 10.3892/etm.2020.8600 PMC7132243 32266033

[21] Zhang X Wang S Lin G Wang D. Down-regulation of circ-PTN suppresses cell proliferation, invasion and glycolysis in glioma by regulating miR-432-5p/RAB10 axis. Neurosci Lett (2020) 735:135153. 10.1016/j.neulet.2020.135153 32629066

[22] Yang Y Li H Liu Y Chi C Ni J Lin X. MiR-4319 hinders YAP expression to restrain non-small cell lung cancer growth through regulation of LIN28-mediated RFX5 stability. Biomed Pharmacother = Biomed Pharmacother (2019) 115:108956. 10.1016/j.biopha.2019.108956 31096145

[23] Chen DB Xie XW Zhao YJ Wang XY Liao WJ Chen P. RFX5 promotes the progression of hepatocellular carcinoma through transcriptional activation of KDM4A. Sci Rep (2020) 10(1):14538. 10.1038/s41598-020-71403-1 32883983 PMC7471945

[24] Huang T Sun L Yuan X Qiu H. Thrombospondin-1 is a multifaceted player in tumor progression. Oncotarget (2017) 8(48):84546–58. 10.18632/oncotarget.19165 PMC5663619 29137447

[25] De Francesco EM Maggiolini M Musti AM. Crosstalk between Notch, HIF-1alpha and GPER in Breast Cancer EMT. Int J Mol Sci (2018) 19(7):2011. 10.3390/ijms19072011 PMC6073901 29996493

[26] Teeuwssen M Fodde R. Wnt Signaling in Ovarian Cancer Stemness, EMT, and Therapy Resistance. J Clin Med (2019) 8(10):11658. 10.3390/jcm8101658 PMC6832489 31614568

[27] Choi JH Hwang YP Kim HG Khanal T Do MT Jin SW. Saponins from the roots of Platycodon grandiflorum suppresses TGFbeta1-induced epithelial-mesenchymal transition via repression of PI3K/Akt, ERK1/2 and Smad2/3 pathway in human lung carcinoma A549 cells. Nutr Cancer (2014) 66(1):140–51. 10.1080/01635581.2014.853087 24341702

[28] Pal SK Nguyen CT Morita KI Miki Y Kayamori K Yamaguchi A. THBS1 is induced by TGFB1 in the cancer stroma and promotes invasion of oral squamous cell carcinoma. J Oral Pathol Med Off Publ Int Assoc Oral Pathol Am Acad Oral Pathol (2016) 45(10):730–9. 10.1111/jop.12430 26850833

[29] Li P Xu T Zhou X Liao L Pang G Luo W. Downregulation of miRNA-141 in breast cancer cells is associated with cell migration and invasion: involvement of ANP32E targeting. Cancer Med (2017) 6(3):662–72. 10.1002/cam4.1024 PMC5345683 28220627

[30] Huang SQ Sun B Xiong ZP Shu Y Zhou HH Zhang W. The dysregulation of tRNAs and tRNA derivatives in cancer. J Exp Clin Cancer Res CR (2018) 37(1):101. 10.1186/s13046-018-0745-z 29743091 PMC5944149

[31] Rashad S Niizuma K Tominaga T. tRNA cleavage: a new insight. Neural Regen Res (2020) 15(1):47–52. 10.4103/1673-5374.264447 31535642 PMC6862408

[32] Zhou K Diebel KW Holy J Skildum A Odean E Hicks DA. A tRNA fragment, tRF5-Glu, regulates BCAR3 expression and proliferation in ovarian cancer cells. Oncotarget (2017) 8(56):95377–91. 10.18632/oncotarget.20709 PMC5707028 29221134

[33] Zhu L Li T Shen Y Yu X Xiao B Guo J. Using tRNA halves as novel biomarkers for the diagnosis of gastric cancer. Cancer Biomarkers Sec A Dis Markers (2019) 25(2):169–76. 10.3233/CBM-182184 PMC13082404 31104009

[34] Li S Shi X Chen M Xu N Sun D Bai R. Angiogenin promotes colorectal cancer metastasis via tiRNA production. Int J Cancer (2019) 145(5):1395–407. 10.1002/ijc.32245 30828790

[35] Deng J Ptashkin RN Chen Y Cheng Z Liu G Phan T. Respiratory Syncytial Virus Utilizes a tRNA Fragment to Suppress Antiviral Responses Through a Novel Targeting Mechanism. Mol Ther J Am Soc Gene Ther (2015) 23(10):1622–9. 10.1038/mt.2015.124 PMC4817927 26156244

[36] Maute RL Schneider C Sumazin P Holmes A Califano A Basso K. tRNA-derived microRNA modulates proliferation and the DNA damage response and is down-regulated in B cell lymphoma. Proc Natl Acad Sci U States A (2013) 110(4):1404–9. 10.1073/pnas.1206761110 PMC3557069 23297232

[37] Muth M Engelhardt BM Kroger N Hussein K Schlue J Busche G. Thrombospondin-1 (TSP-1) in primary myelofibrosis (PMF) - a megakaryocyte-derived biomarker which largely discriminates PMF from essential thrombocythemia. Ann Hematol (2011) 90(1):33–40. 10.1007/s00277-010-1024-z 20625903

[38] Wynn TA. Common and unique mechanisms regulate fibrosis in various fibroproliferative diseases. J Clin Invest (2007) 117(3):524–9. 10.1172/JCI31487 PMC1804380 17332879

[39] Mondet J Hussein K Mossuz P. Circulating Cytokine Levels as Markers of Inflammation in Philadelphia Negative Myeloproliferative Neoplasms: Diagnostic and Prognostic Interest. Mediators Inflamm (2015) 2015:670580. 10.1155/2015/670580 26525644 PMC4617441

[40] Isenberg JS Roberts DD. THBS1 (thrombospondin-1). Atlas Genet Cytogenet Oncol Haemat (2020) 24(8):291–9. 10.4267/2042/70774 PMC7687907 33244322

[41] Tsuchida R Osawa T Wang F Nishii R Das B Tsuchida S. BMP4/Thrombospondin-1 loop paracrinically inhibits tumor angiogenesis and suppresses the growth of solid tumors. Oncogene (2014) 33(29):3803–11. 10.1038/onc.2013.358 24013228

[42] Shen J Cao B Wang Y Ma C Zeng Z Liu L. Hippo component YAP promotes focal adhesion and tumour aggressiveness via transcriptionally activating THBS1/FAK signalling in breast cancer. J Exp Clin Cancer Res CR (2018) 37(1):175. 10.1186/s13046-018-0850-z 30055645 PMC6064138

[43] Sartor MA Mahavisno V Keshamouni VG Cavalcoli J Wright Z Karnovsky A. ConceptGen: a gene set enrichment and gene set relation mapping tool. Bioinformatics (2010) 26(4):456–63. 10.1093/bioinformatics/btp683 PMC2852214 20007254

[44] Koinuma D Tsutsumi S Kamimura N Taniguchi H Miyazawa K Sunamura M. Chromatin immunoprecipitation on microarray analysis of Smad2/3 binding sites reveals roles of ETS1 and TFAP2A in transforming growth factor beta signaling. Mol Cell Biol (2009) 29(1):172–86. 10.1128/MCB.01038-08 PMC2612478 18955504

[45] Nakagawa T Lan HY Glushakova O Zhu HJ Kang DH Schreiner GF. Role of ERK1/2 and p38 mitogen-activated protein kinases in the regulation of thrombospondin-1 by TGF-beta1 in rat proximal tubular cells and mouse fibroblasts. J Am Soc Nephrol JASN (2005) 16(4):899–904. 10.1681/ASN.2004080689 15716330

[46] Cui W Zhou J Dehne N Brune B. Hypoxia induces calpain activity and degrades SMAD2 to attenuate TGFbeta signaling in macrophages. Cell Biosc (2015) 5:36. 10.1186/s13578-015-0026-x PMC4491253 26146544

