# Supplementary material for: tRNA-Derived Fragment tRF-17-79MP9PP Attenuates Cell Invasion and Migration via THBS1/TGF-β1/Smad3 Axis in Breast Cancer
Source: Front Oncol. 2021 Apr 12;11:656078. doi: 10.3389/fonc.2021.656078 (PMC8072113; doi:10.3389/fonc.2021.656078)
Supplement: Supplementary file 1 [file Table_1.docx]

**Supplementary Table 1** Clinical-pathological information of serum samples

| Characteristics | Breast cancer | Benign breast disease | Healthy control |
| --- | --- | --- | --- |
|  | No (%) | No (%) | No (%) |
| Number | 76 61.78 | 20 16.26 | 27 21.96 |
| Age (years) |  |  |  |
| Mean ± SD | 50.51 ± 10.06 | 43.33 ±14.01 | 48.95 ± 11.92 |
| Benign breast disease |  |  |  |
| Hyperplasia |  | 9 45.00 |  |
| Fibroadenoma |  | 9 45.00 |  |
| Breast nodule |  | 2 10.00 |  |
| Tumor stage |  |  |  |
| I | 11 14.47 |  |  |
| II | 25 32.89 |  |  |
| III | 28 36.84 |  |  |
| IV | 12 15.80 |  |  |
| Lymph node metastasis |  |  |  |
| No | 38 50.00 |  |  |
| Yes | 38 50.00 |  |  |
