# Supplementary material for: tRNA-Derived Fragment tRF-17-79MP9PP Attenuates Cell Invasion and Migration via THBS1/TGF-β1/Smad3 Axis in Breast Cancer
Source: Front Oncol. 2021 Apr 12;11:656078. doi: 10.3389/fonc.2021.656078 (PMC8072113; doi:10.3389/fonc.2021.656078)
Supplement: Supplementary file 2 [file Table_2.docx]

**Supplementary Table 2** Clinical-pathological information of tissue samples

| Characteristics | Number of patients | % |
| --- | --- | --- |
| Age(years) |  |  |
| Mean ± SD | 52.92 ±10.54 |  |
| Tumor stage |  |  |
| I | 0 |  |
| II | 7 | 43.75 |
| III | 9 | 56.25 |
| IV | 0 |  |
| Lymph node metastasis |  |  |
| No | 10 | 62.50 |
| Yes | 6 | 37.50 |
