# Supplementary material for: tRNA-Derived Fragment tRF-17-79MP9PP Attenuates Cell Invasion and Migration via THBS1/TGF-β1/Smad3 Axis in Breast Cancer
Source: Front Oncol. 2021 Apr 12;11:656078. doi: 10.3389/fonc.2021.656078 (PMC8072113; doi:10.3389/fonc.2021.656078)
Supplement: Supplementary file 3 [file Table_3.docx]

**Supplementary Table 3** Primers used in the polymerase chain reaction

| Primer names | Primer sequences (5'-3') |
| --- | --- |
| THBS1 forward | AGACTCCGCATCGCAAAGG |
| THBS1 reverse | TCACCACGTTGTTGTCAAGGG |
| KIF3A forward | GTGTTCGAGCTATTCCTGAACTT |
| KIF3A reverse | CCTCTAACCTTTGTGTCTGATCC |
| RAB10 forward | GAGTTGGCCGTAGTGAGAGG |
| RAB10 reverse | AGGTCGTACGTCTTCTTCGC |
| RFX5 forward | GATGAGCCTGATGCTAAGAGC |
| RFX5 reverse | CCCTCTACTTTGTTCTGCACG |
| tRF-17-79MP9PP forward | TCTACAGTCCGACGATCGTTTC |
| tRF-17-79MP9PP reverse | TGCTCTTCCGATCTCACTACACTA |
| U6 forward | GCTTCGGCAGCACATATACTAAAAT |
| U6 reverse | CGCTTCACGAATTTGCGTGTCAT |
| β-actin forward | TGGCACCCAGCACAATGAA |
| β-actin reverse | CTAAGTCATAGTCCGCCTAGAAGCA |
